# Pas de Trois: An Overview of Penta-, Tetra-, and Octo-Tricopeptide Repeat Proteins From *Chlamydomonas reinhardtii* and Their Role in Chloroplast Gene Expression

**DOI:** 10.3389/fpls.2021.775366

**Published:** 2021-11-17

**Authors:** Karla S. Macedo-Osorio, Agustino Martínez-Antonio, Jesús A. Badillo-Corona

**Affiliations:** ^1^Instituto Politécnico Nacional, Unidad Profesional Interdisciplinaria de Biotecnología, México City, México; ^2^Biological Engineering Laboratory, Genetic Engineering Department, Centro de Investigación y Estudios Avanzados del Instituto Politécnico Nacional-Unidad Irapuato, Irapuato, México; ^3^División de Ciencias Biológicas y de la Salud, Universidad Autónoma Metropolitana-Xochimilco, México City, México

**Keywords:** helical repeat proteins, PPR, OPR, TPR, HAT-TPR, chloroplast mRNA-processing, anterograde control

## Abstract

Penta-, Tetra-, and Octo-tricopeptide repeat (PPR, TPR, and OPR) proteins are nucleus-encoded proteins composed of tandem repeats of 35, 34, and 38–40 amino acids, respectively. They form helix-turn-helix structures that interact with mRNA or other proteins and participate in RNA stabilization, processing, maturation, and act as translation enhancers of chloroplast and mitochondrial mRNAs. These helical repeat proteins are unevenly present in plants and algae. While PPR proteins are more abundant in plants than in algae, OPR proteins are more abundant in algae. In *Arabidopsis*, maize, and rice there have been 450, 661, and 477 PPR proteins identified, respectively, which contrasts with only 14 PPR proteins identified in *Chlamydomonas reinhardtii*. Likewise, more than 120 OPR proteins members have been predicted from the nuclear genome of *C. reinhardtii* and only one has been identified in *Arabidopsis thaliana*. Due to their abundance in land plants, PPR proteins have been largely characterized making it possible to elucidate their RNA-binding code. This has even allowed researchers to generate engineered PPR proteins with defined affinity to a particular target, which has served as the basis to develop tools for gene expression in biotechnological applications. However, fine elucidation of the helical repeat proteins code in Chlamydomonas is a pending task. In this review, we summarize the current knowledge on the role PPR, TPR, and OPR proteins play in chloroplast gene expression in the green algae *C. reinhardtii*, pointing to relevant similarities and differences with their counterparts in plants. We also recapitulate on how these proteins have been engineered and shown to serve as mRNA regulatory factors for biotechnological applications in plants and how this could be used as a starting point for applications in algae.

## Introduction

Evolutionary biologists estimate that it was 1.5 billion years ago when plastids started to develop as the result of the engulfment of a cyanobacterium by a heterotrophic eukaryotic cell (Yoon et al., 2004). Later, it is widely accepted, this endosymbiosis process gave rise to chloroplasts in plants and algae (reviewed by Stadnichuk and Kusnetsov, 2021). The evolution of this endosymbiosis resulted in a drastic genome reduction of the originally engulfed cyanobacterium; 0.15 Mbp presently in chloroplasts vs. 3 Mbp in free-living cyanobacteria (Martin et al., 2002; Yagi and Shiina, 2014). While thousands of genes disappeared, most likely as the result of being redundant with the ones already present in the host, others were transferred and integrated into the host nuclear genome (Bock, 2017; De Marchis et al., 2019). As a result of this genome reduction, 2–5% of the genes found in free-living cyanobacteria remain in modern chloroplasts (Eckardt, 2006; Stegemann and Bock, 2006; Yagi and Shiina, 2014). This gene interchange is also made evident by the fact that 3.5–6% of protein-coding genes in the nuclear genome of Chlamydomonas and other Plantae have a cyanobacterial origin (Moustafa and Bhattacharya, 2008). This genetic rearrangement ultimately led to the chloroplasts as we know them today: semi-autonomous organelles that contain their own genome and the expression machinery for many genes involved in protein synthesis (including tRNAs and rRNAs) and photosynthesis (including proteins involved in light capture, CO_2_ fixation, and ATP synthesis).

It has been estimated that around 93–99% of proteins functioning inside chloroplasts are encoded in the nucleus (Woodson and Chory, 2008), translated in the cytosol, and then transported into the chloroplast (Nakai, 2018). In photosynthetic organisms, the subunits of all photosynthetic complexes, the translation apparatus, some proteins of the chloroplast envelope, and the key enzyme in carbon fixation, RuBisCo, are encoded by genes located in both the nuclear and chloroplast genomes (Börner, 2017). For example, in *Chlamydomonas reinhardtii*, Photosystem I (PSI) is composed of 14 proteins, four of which are encoded in the chloroplast (PsaA, PsaB, PsaC, and PsaJ subunits) and the other 10 are nucleus-encoded (Harris, 2009). The assembly of the protein complexes of the photosystems I and II, the ATP synthase, cytochrome *b*_***6***_*f* complex, and RuBisCo is regulated by the control by epistasis of synthesis (CES) process, dependent on the regulation of translation. The synthesis of one subunit becomes dependent on the synthesis of another: when certain core subunits are absent, synthesis of other chloroplast-encoded subunits from the same protein complex is reduced (Choquet et al., 1998; Choquet and Wollman, 2009). Similarly, the cofactors that are accumulated in excess of their binding proteins can regulate their own synthesis and that of the proteins (Pogson et al., 2008). The expression of the divided encoded genetic information requires coordinated communication between chloroplast and nucleus to maintain cell homeostasis.

Cellular processes such as chloroplast biogenesis (reviewed by Jarvis and López-Juez, 2013; Yoo et al., 2020), plastid differentiation (reviewed by Sadali et al., 2019), photosynthesis including the assembly of photosynthetic apparatus and expression of photosynthetic genes (Lefebvre-Legendre et al., 2015, 2016; Douchi et al., 2016; Krupinska et al., 2019), pigment production (Terry and Bampton, 2019; Ganusova et al., 2020), and other metabolic processes such as starch and lipid biosynthesis, cell wall synthesis and/or modification, sugar transport, stress responses, and reactive oxygen species (ROS) responses are highly dependent on communication between the nucleus and the chloroplast (reviewed by Woodson and Chory, 2008; Börner, 2017; Piñas-Fernández and Strand, 2018). ***Trans***-acting regulatory factors are generated in the nucleus and are essential for transcriptional, post-transcriptional, and translational regulation of chloroplast gene expression (Woodson and Chory, 2008; Piñas-Fernández and Strand, 2018). The regulation from the nucleus to the chloroplast is known as anterograde signaling. In contrast, retrograde signaling is when the signals originate from the organelles (chloroplasts and mitochondria) to influence the expression of nuclear genes, some of which, in turn, can alter the anterograde control (Hernández-Verdeja and Strand, 2018). Synchronization between anterograde and retrograde signaling allows the cell to survive against changes in the environment (Rochaix and Ramundo, 2017). These changes include temperature, light intensity, drought, and nutrient deficiency (Sun and Guo, 2016; Rochaix and Ramundo, 2017). As part of the anterograde signaling, in higher plants and microalgae, many nucleus-encoded proteins are RNA-binding factors for post-transcriptional regulation of gene expression, such as RNA stabilization, splicing, intercistronic processing of polycistronic RNAs, editing of chloroplast transcripts, and regulation of the translation (Lyska et al., 2013; Manavski et al., 2018; Zoschke and Bock, 2018; De Marchis et al., 2019).

RNA-binding proteins involved in post-transcriptional gene expression include the helical repeat protein family. This superfamily comprises the Tetra-tricopeptide repeat (TPR), Half-a-tetratricopeptide repeat (HAT), Penta-tricopeptide repeat (PPR), and Octo-tricopeptide repeat (OPR) proteins. TPR proteins are present in prokaryotic and eukaryotic organisms, where their principal function is the regulation of many cellular processes, including translation, by mediating protein–protein interactions (Bohne et al., 2016). PPR proteins are found in all eukaryotic organisms. In land plants, hundreds of PPR proteins have been identified, many of them even functionally characterized, determining that they are involved in RNA metabolism and translation regulation of organellar genes (Shikanai and Fujii, 2013; Barkan and Small, 2014; Rovira and Smith, 2019). OPR proteins are also found in prokaryotes and eukaryotes and similar functions have been attributed to them; however, although the number of OPR proteins is limited in land plants, in algae it is quite large (Bohne et al., 2009; Rahire et al., 2012).

Due to its ease of cultivation and its relative simplicity, the microalga *C. reinhardtii* is one of the best model organisms for the study of various cellular processes, including photosynthesis, flagellar function and pigment, biofuel, and recombinant proteins production (Salomé and Merchant, 2019). Furthermore, the study of the mechanisms regulating chloroplast gene expression in *C. reinhardtii* is crucial for the development of tools for the improvement of the production of biomass and bioactive compounds. In this paper, we first review the role of PPR, TPR (including HAT), and OPR proteins in chloroplast gene expression in *C. reinhardtii* and then consider the emerging tools being developed for biotechnological applications.

### Chloroplast Gene Expression

In the last decade, the study of chloroplast gene function and regulation gained great attention as this organelle showed to be a low-cost platform for recombinant protein production (Economou et al., 2014; Shamriz and Ofoghi, 2017; Dyo and Purton, 2018). In the chloroplast of the unicellular microalga *C. reinhardtii* more than 100 recombinant proteins, including antibodies (Mayfield et al., 2003; Tran et al., 2009), immunotoxins (Tran et al., 2013a,b), antigens (Dreesen et al., 2010; Michelet et al., 2011; Jones et al., 2013), toxins (Kang et al., 2017), and growth factors (Rasala et al., 2010; Wannathong et al., 2016) have been produced. This has been possible thanks to advances in the study of chloroplast functioning and the development of molecular tools. The plastid genome was sequenced almost 2 decades ago and consists of 99 genes that include tRNAs, rRNAs, plastid-encoded RNA polymerase subunits, ribosomal and photosynthetic proteins (Maul et al., 2002; Gallaher et al., 2018). The synthesis of chloroplast proteins is a complex process highly regulated at translational level but also depending on changes in transcription rate and mRNA degradation (Herrin, 2009). Although, chloroplast mRNAs do not possess a 5'trimethylguanosine cap like cytoplasmic mRNAs, they have a relatively long half-life in the range of minutes to hours (Herrin, 2009). In many cases, the stability of these transcripts depends on nucleus-encoded proteins that protect and prevent mRNA degradation by nucleases.

**Figure 1:**
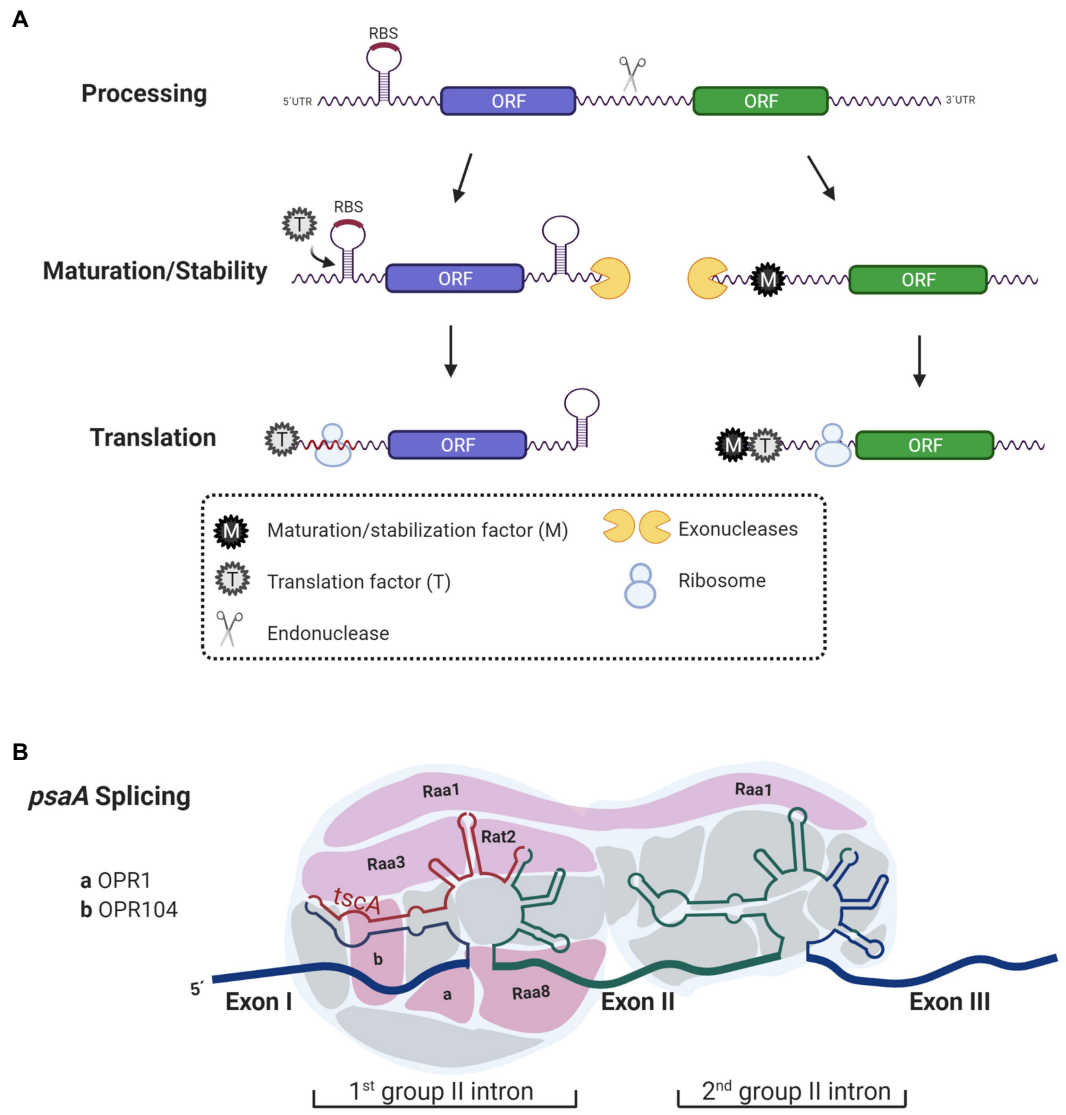
Functions of Octo-, Tetra-, and Pentra-tricopeptide repeat (PPR) proteins. **(A)** Octo-tricopeptide repeat (OPR), tetra-tricopeptide repeat (TPR)-half-a-tetratricopeptide repeat (HAT), and PPR proteins act as maturation/stabilization (M) and translation factors (T). Before being translated, polycistronic transcripts are processed into monocistronic units by an endonuclease. The 5'- and 3'-ends generated after the cleavage are exposed and prone to degradation by exonucleases that trim RNA-ends until they are blocked by secondary structures in the mRNA or by RNA binding proteins, like OPR, HAT, and PPR proteins acting as M factors. Additionally, some transcripts need T factors (T) to promote and enhance translation. Some T factors anchor to mRNA and unmask ribosome binding sites (RBS) thus changing the secondary structure in the mRNA to facilitate ribosome biding to the mRNA. Some other T factors bind further away from the RBS and their mode of action activating translation is still unknown. **(B)** Some OPR proteins have been proposed to act as interaction modules for RNA processing. Some of the nucleus-encoded factors [RAA1, RAA3, RAT2, RAA8, OPR1 (Cre01.g001501), and OPR104 (Cre17.g698750)] that participate in the splicing of *psaA* that contain OPR motifs are shown. These OPR proteins interact with *psaA* mRNA facilitating splicing.

For many years, chloroplast gene arrangement was thought to result in the transcription of mono, bi, or tri-cistronic units. However, it was recently reported that 70% of genes from *C. reinhardtii* chloroplast are co-transcribed forming long polycistronic units, although it is still clear that in some cases, some of these genes are also regulated by single promoters (Cavaiuolo et al., 2017; Gallaher et al., 2018). Polycistronic mRNAs are integrated by two or more cistrons transcribed from the same promoter, however, unlike prokaryotes, cistron units in chloroplast are separated by intergenic regions rich in A–U (Pfalz et al., 2009). Before translation, polycistronic transcripts must be processed; intergenic regions serve as the target of endonucleases to adjust polycistronic mRNAs into translatable monocistrons. Once cistrons are cleaved, the resulting transcripts are exposed to exonucleases, which degrade them further from their ends until degradation is blocked by an internal robust mRNA-structure or by an mRNA-binding protein (RBP) bound to the transcript (Pfalz et al., 2009; Prikryl et al., 2011; Stoppel and Meurer, 2012). mRNA-binding proteins anchor to either the 5'-untranslated region (UTR) or 3'-UTR of the transcript protecting it from degradation during the trimming process by exonucleases (Figure 1A). In photosynthetic genes of plants and microalgae, these protective functions have been experimentally confirmed to be attributed to members of the helical repeat protein family: namely, PPR, OPR, or TPR proteins (Boudreau et al., 2000; Loiselay et al., 2008; Pfalz et al., 2009; Johnson et al., 2010; Wang et al., 2015; Viola et al., 2019). In plants and Chlamydomonas, PPR and OPR proteins act as post-transcriptional factors that participate in RNA stabilization and translation of chloroplast transcripts (Johnson et al., 2010; Prikryl et al., 2011; Wang et al., 2015; Viola et al., 2019). Although some TPR proteins also participate in these processes, their main function relies on the protein–protein interactions required for the assembly of photosynthesis complexes (Bohne et al., 2016). Additionally, in mitochondria and chloroplast of plants, PPR proteins also have editing functions (Andrés-Colás et al., 2017; Guillaumot et al., 2017).

P/O/TPR proteins are nucleus-encoded proteins characterized by the presence of tandem arrays of degenerated 35, 38–40, and 34 amino acid repeats, respectively (Rovira and Smith, 2019). On the one hand, PPR proteins are more abundant in plants than in microalgae. In *C. reinhardtii*, 14 PPR proteins have been identified (Tourasse et al., 2013). By contrast, in the genomes of *A. thaliana*, maize, and rice approximately 450, 661, and 477 members of the PPR proteins family have been identified, respectively.^1^ Most of these PPR proteins have been predicted to be functional in plastids and mitochondria (Gutmann et al., 2019; Rovira and Smith, 2019). On the other hand, OPR proteins are disproportionally more abundant in microalgae where around >120 OPR have been estimated to be encoded in the nuclear genome of *C. reinhardtii* (Cavaiuolo et al., 2017; Chlamydomonas genome; https://phytozome-next.jgi.doe.gov/info/Creinhardtii_v5_6) vs. only one identified in *A. thaliana* (Kleinknecht et al., 2014). Although both PPR and OPR proteins seem to have similar functions associated with stability, maturation, and translation of chloroplast and mitochondria transcripts, the difference in their distribution in algae and plants may indicate different evolutionary routes in the regulation of gene expression in the organelles of these lineages (Fujii and Small, 2011; Rovira and Smith, 2019).

According to the nomenclature established for the Organelle *Trans*-Acting Factors (OTAFs) in *C. reinhardtii*, P/O/TPR proteins are named in their first letter according to their function: **M** if they are maturation or stability factors required for the stable accumulation of their target mRNA; or **T**, if they are factors required for translation of specific transcripts, respectively (Cavaiuolo et al., 2017). They are then assigned a letter A, B, C, D, or R if their targets are mRNAs for PSI, PSII, cytochrome *b_6_f*, ATP synthase, and RuBisCO subunits, respectively. A third letter corresponding to the last letter of the name of their target gene is required to be incorporated (Wang et al., 2015). For instance, MAC1 is a **M**aturation factor for the *psaC* mRNA that in turn is translated into the PsaC subunit from PSI. Similarly, TAB1 is required as a **T**ranslation factor for the production of PsaB protein of PSI.

As it was already observed, the regulation of chloroplast gene expression relies on several layers of control. One of these layers is the result of the coordinated action of P/O/TPR proteins as we have glimpsed. In the following sections, we explore specific roles and mechanisms of action of P/O/TPR proteins in *C. reinhardtii*.

## Penta-Tricopeptide Repeat Proteins

Penta-tricopeptide Repeat proteins are distributed in all eucaryotes, and due to their abundance and relevant functions, these proteins have been extensively studied in plants (Gutmann et al., 2019). These were initially identified in nuclear mutants with non-photosynthetic phenotypes and altered post-transcriptional processes (Fisk et al., 1999). Shortly after, the sequence of the *A. thaliana* genome revealed a new protein family that comprised hundreds of genes that until that moment had an unknown function (Aubourg et al., 2000). Later, Small and Peeters (2000) named them, PPR Proteins following their structural similarity with the previously described TPR motifs. Interestingly and surprisingly, Lurin et al. (2004) predicted that two-thirds of these proteins are targeted to mitochondria or chloroplasts in Arabidopsis.

**Figure 2:**
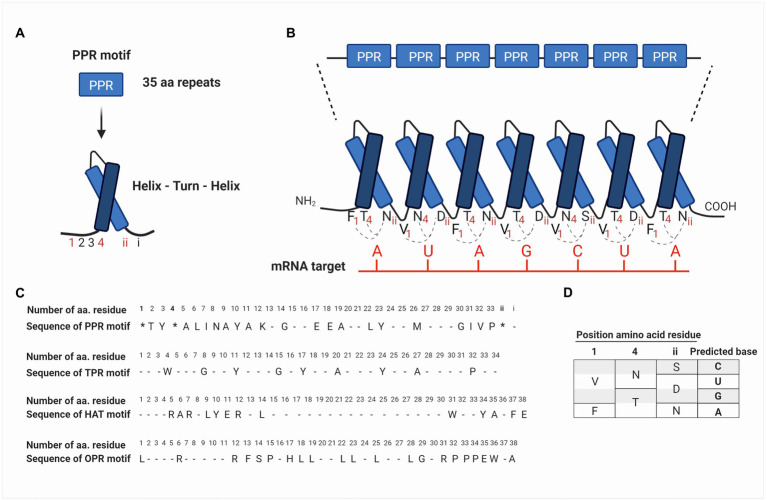
Schematic representation of the structural arrangement of PPR proteins and their binding sites to mRNA targets based on the PPR code. **(A)** PPR proteins are composed of repeats of degenerate motifs of 35 amino acids. The PPR motif forms two antiparallel α-helices, which interact to produce a helix-turn-helix motif. Amino acids in the PPR motif are identified from 1 to 33 according to their position starting from the NH_4_-terminus. The last two amino acids are identified as i (last residue) and ii (penultimate residue), according to the position starting from the COOH-terminus (see also **C** in this figure). **(B)** PPR motifs are repeated in tandem and constitute a PPR protein, where each motif interacts with one ribonucleotide on the mRNA target. **(C)** The PPR motif identified for first time in *Arabidopsis thaliana* by Small and Peeters (2000) is shown at the top, followed by the consensus sequences of TPR and HAT motifs identified in proteins of *Chlamydomonas reinhardtii*, *Physcomitrella patens*, and *A. thaliana* (Bohne et al., 2016). At the bottom, the OPR motifs identified in TCB2 protein (Auchincloss et al., 2002), which contains the consensus sequence PPPEW present in most of OPR proteins is also shown. In the PPR motif, amino acid residues at positions 1, 4, and ii (indicated with an asterisk *) are determinants in the recognition of RNA bases. Conserved residues are indicated with the consensus one letter amino acid code, while degenerated residues are indicated with a dash. **(D)** Summary of the current PPR code proposed by Barkan et al. (2012) and Yagi and Shiina (2014). Different combinations of amino acids at positions 1, 4, and ii result in a code specific for a certain RNA base. For instance, Val (V), Asn (N), and Ser (S) at positions 1, 4, and ii, respectively, can bind cytosine in the mRNA target. Similarly, Phe (F), Thr (T), and Asn (N) bind adenine.

These nucleus-encoded proteins are composed of degenerated motifs of 35 amino acids repeated in tandem (Small and Peeters, 2000). The PPR motif forms two antiparallel α-helices, which interact to produce a helix-turn-helix motif (Figures 2A,B). The series of helix-turn-helix motifs form an α-solenoid structure that interacts with nucleic acids and/or with other proteins (Barkan and Small, 2014; Wang et al., 2015). The bioinformatic analysis had predicted that each PPR-motif interacts with one ribonucleotide of their target RNA and that amino acids in precise positions determine the ribonucleotide-binding specificity (Figures 2B–D; Barkan et al., 2012; Shen et al., 2016).

In *C. reinhardtii* 14 PPR proteins have been identified (Tourasse et al., 2013), but only four have been experimentally characterized (Loiselay et al., 2008; Johnson et al., 2010; Jalal et al., 2015; Cavaiuolo et al., 2017). All four have been determined to be localized in the chloroplast and are involved in the stabilization, maturation, and/or translation of photosynthetic transcripts (Loiselay et al., 2008; Johnson et al., 2010; Jalal et al., 2015; Table 1). The first PPR protein identified in Chlamydomonas was MCA1 (PPR14), this protein binds to 21 nucleotides at the 5′-terminus of the chloroplast *petA* mRNA, protecting it from degradation by exonucleases and in consequence facilitating the synthesis of cytochrome *f* (Loiselay et al., 2008). MRL1 (Maturation factor of *rbcL*; PPR2), was the second PPR protein identified, and it is conserved in *C. reinhardtii* and *Arabidopsis*; this protein is involved in regulating RuBisCo large subunit (*rbcL*) transcript levels (Johnson et al., 2010). MRL1 binds to the 5′ end of the *rbcL* mRNA, stabilizing and protecting it from exonucleases degradation. Mutants deficient in MRL1 are deficient in RuBisCO large subunit, which results in a lack of the RuBisCO holoenzyme and thus yield a non-photosynthetic phenotype (Johnson et al., 2010; Johnson, 2011). Curiously, *Arabidopsis mrl1* mutants still accumulate the unprocessed *rbcL* mRNA, can synthesize RuBisCo, and grow normally under photoautotrophic conditions. TCB1 (PPR1) is the ortholog of HCF152 from plants. Chlamydomonas mutants of *ppr1* are non-phototrophic and the phenotype resembles that of cytochrome *b*_6_*f* mutants. From small RNA profiling, it has been proposed that the role of PPR1 is mainly as a translation factor, hence the TCB1 name, and that in addition, it stabilizes *petB* (Cavaiuolo et al., 2017).

**Table 1:** Penta-tricopeptide repeat proteins identified and studied in *C. reinhardtii.*

Name	Number of PPR motifs	Target	Function	Reference
TCB1 (PPR1)	11*	*petB*	Translation and stabilization of *petB* mRNA	Tourasse et al., 2013*; Cavaiuolo et al., 2017
MRL1 (PPR2)	12/9*	*rbcL*	Stabilization of *rbcL* mRNA	Johnson et al., 2010; Johnson, 2011; Tourasse et al., 2013*
PPR3	9	*rps4*, target suggested by the PPR code	Not studied yet	Tourasse et al., 2013; Cavaiuolo et al., 2017
PPR4	10	Unknown	Not studied yet	Tourasse et al., 2013
PPR5	7	Unknown	Not studied yet	Tourasse et al., 2013
PPR6	10	*psbF*, target suggested by the PPR code	Not studied yet	Tourasse et al., 2013; Cavaiuolo et al., 2017
PPR7	2*/4	*rbcL, rpoC2, psbH, tscA,* 16S *rRNA, psbJ*, and *atpA* gene clusters	Stabilization of *rbcL*, *rpoC2*, *psbH*, and *tscA* transcripts. Maturation of polycistronic mRNAs *psbJ* and *atpA* gene clusters as well as of 16S rRNA.	Tourasse et al., 2013*; Jalal et al., 2015
PPR8	8	Unknown	Not studied yet	Tourasse et al., 2013
PPR9	4	Unknown	Not studied yet	Tourasse et al., 2013
PPR10	5	Unknown	Not studied yet	Tourasse et al., 2013
PPR11	2	Unknown	Not studied yet	Tourasse et al., 2013
PPR12	2	Unknown	Not studied yet	Tourasse et al., 2013
PPR13	5	Unknown	Not studied yet	Tourasse et al., 2013
MCA1 (PPR14)	14	*petA*	Stabilization of *petA* mRNA	Raynaud et al., 2007; Loiselay et al., 2008; Boulouis et al., 2011; Tourasse et al., 2013

Asterisks (^*^) indicate the specific reference of that data.

In contrast to MCA1 and MRL1, both of which have a single mRNA target, PPR7 protein, which is the most highly expressed PPR protein in Chlamydomonas, participates in regulating the expression of several chloroplast genes (Jalal et al., 2015). PPR7 is part of a ribonucleoprotein complex that contributes to the stabilization of *rbcL*, *rpoC2*, *psbH*, and *tscA* transcripts (Jalal et al., 2015). Additionally, PPR7 also participates in the processing and maturation of the following polycistronic transcripts: the gene cluster *atpA*-*psbI*-*cemA*-*atpH*, which encodes for the α-subunit of the ATP synthase; a small PSII subunit, a putative envelope membrane protein involved in inorganic carbon uptake; and the subunit III of chloroplast ATP synthase, respectively (Jalal et al., 2015). PPR7 also participates in the maturation of the co-transcript *psbJ-atpI-psaJ-rps12*, which encodes for PsbJ (a PSII reaction center protein), the subunit IV of ATP synthase; PsaJ (a PSI reaction center subunit), and the ribosomal protein S12. Additionally, PPR7 participates in processing the 16S rRNA, which is part of the *rrn* operon that encodes for the 16S, 7S, 3S, 23S, 5S rRNAs, and two tRNAs (*trnI* and *trnA*; Jalal et al., 2015). Detailed mechanisms of PPR7 in processing all these polycistronic transcripts are still scarce and unclear, particularly when compared to the level of details known for PPR proteins in plants (Pfalz et al., 2009; Stoppel and Meurer, 2012). A hypothesis is that PPR7 could recruit certain endonucleases or modify large secondary structures in large mRNAs to unmask nuclease sensitive targets. In any case, given the diversity of functions of the regulated genes, PPR7 is essential for the proper functioning of chloroplasts (Jalal et al., 2015).

## Tetra-Tricopeptide Repeat Proteins

Like PPR proteins, TPR proteins are helical repeat proteins characterized by the presence of 1–26 tandem repeats, each one of 34 amino acids that form an antiparallel α-helical hairpin (Bohne et al., 2016). The TPR motifs function as a scaffold for protein assembly in multi-subunit complexes and are involved in various processes ranging from transcriptional regulation and RNA metabolism to protein folding, chlorophyll synthesis, and transport (Table 2). Given the diversity of functions, it is not surprising that the location of TPR proteins is not limited to the chloroplast or the mitochondria. Two flagellar subunit proteins with TPR motifs, IFT139 and IFT88, have been identified in *C. reinhardtii* as part of the IFT-A and IFT-B subcomplex involved in intraflagellar transport (Pazour et al., 2000; Snell and Goodenough, 2009; Behal et al., 2012). IFT88-deficient *C. reinhardtii* mutants show a phenotype without flagella (Pazour et al., 2000). Because IFT88 is conserved in green algae, nematodes, and vertebrates, the analysis of *C. reinhardtii* IFT88 mutants has allowed the study of intra-flagellar transport in other species, for example, the study of its homologous genes in mice and humans called Tg737. Mice with Tg737 defects die shortly after birth from polycystic kidney disease (Pazour et al., 2000).

**Table 2:** Tetra-tricopeptide repeat and HAT-TPR proteins identified and studied in *C. reinhardtii.*

Type of protein	Name	Number of TPR motifs	Target	Function	Reference
TPR	CGL71	3	Not apply	Assembly of the PSI complex. Involved in protecting the PSI assembly process from oxidative disruption	Heinnickel et al., 2016
	IFT139	4	Not apply	Involved in the intraflagellar transport. IFTA subcomplex.	Pazour et al., 2000; Behal et al., 2012
	IFT88	2	Not apply	Involved in the intraflagellar transport. IFTB subcomplex.	Pazour et al., 2000; Behal et al., 2012
	FAP70	2	Not apply	Involved in ciliary motility	Shamoto et al., 2018; Hou et al., 2021
	FLP	2	Not apply	Regulator of the chlorophyll synthesis pathway	Falciatore et al., 2005
	REP27	2	*psbA*	Factor for the *psbA* mRNA translation initiation and assembly of the nascent D1 protein. Activation of the bound D1 in the PSII reaction center during the PSII repair process.	Park et al., 2007; Dewez et al., 2009
	Tic40	1	Not apply	Component of the chloroplast protein import machinery.	Kalanon and McFadden, 2008
	Ycf3	3	PsaA, PsaD subunits	Assembly of the PSI complex.	Boudreau et al., 1997; Naver et al., 2001; Rochaix et al., 2004; Nellaepalli et al., 2018
HAT-TPR	MAC1	12	*psaC*	Stabilization of *psaC* 5'UTR.	Douchi et al., 2016
	MBB1	10	*psbB-psbT-psbH*	Processing of *psbB-psbT-psbH* operon. Stabilization of *psbB-psbT* and *psbH* 5'UTR.	Monod et al., 1992; Vaistij et al., 2000a,b; Loizeau et al., 2014
	MBD1 (NAC2)	9	*psbD*	Stabilization of *psbD* 5'UTR.	Kuchka et al., 1989; Boudreau et al., 2000; Schwarz et al., 2007, 2012

Tetra-tricopeptide repeat motifs can also act as sites for protein–protein interactions; in Chlamydomonas and tobacco, Ycf3, a chloroplasts-encoded TPR protein, is responsible for the initial assembly of PSI (Boudreau et al., 1997). The Ycf3 protein interacts with the PsaA and PsaB subunits forming the PSI reaction center subcomplex to which the Ycf4-module binds to provide stabilization and to facilitate the integration of the other central PSI subunits (PsaC, D, E, F, H, I, J, and L) to form the mature PSI–LHCI supercomplex (Naver et al., 2001; Nellaepalli et al., 2018, 2021). Ycf3 mutants display a light-sensitive phenotype, and they cannot grow photoautotrophically, demonstrating that Ycf3 is essential for photosynthesis. In addition to Ycf3, CGL71, another TPR protein, also participates in the biogenesis and stability of PSI, protecting it from oxidative disruption during its assembly (Heinnickel et al., 2016). FLP and Tic40 also participate in chloroplast biogenesis, the former in the regulation of chlorophyll biosynthesis and the latter as a component of the chloroplast import machinery (Falciatore et al., 2005; Kalanon and McFadden, 2008).

Another example is REP27, a TPR protein located on the thylakoid membrane, which participates in the PSII repair cycle (Park et al., 2007; Dewez et al., 2009). During oxygenic photosynthesis, the abundance of O_2_ and the formation of oxidants lead to photo-oxidative damages causing irreversible inactivation of the D1 protein, impairing electron transport, and inhibiting the function of PSII (Park et al., 2007). REP27 participates by facilitating and regulating different stages of D1 biosynthesis *de novo*. It first acts as a factor for the initiation of *psbA* translation (D1 protein). Then it facilitates the assembly of the nascent D1 peptide. Finally, REP27 activates the D1 protein through post-translational modifications conferring it its functional state in the PSII complex (Dewez et al., 2009). Thus, REP27 plays a triple role, in the regulation of PSII turnover, assembly, and activation.

Like PPR proteins, TPR proteins are also involved in the metabolism of RNA, and this function has been associated with the HAT (half-a-TPR) motif, a subclass of TPR motifs found in several proteins that stabilize and/or activate the translation of specific chloroplast RNAs (Bohne et al., 2016). Unlike the TPR motif, the HAT motifs are characterized by a distinct pattern of conserved amino acid positions and by the presence of highly conserved aromatic residues, mainly tyrosine and tryptophan (Preker and Keller, 1998). In *C. reinhardtii* the existence of 10 HAT proteins has been estimated (Table 2; Bohne et al., 2016), from which three have been functionally characterized; NAC2 (also named MBD1; Kuchka et al., 1989, Boudreau et al., 2000; Schwarz et al., 2007), MAC1 (Douchi et al., 2016), and MBB1 (Vaistij et al., 2000a,b; Loizeau et al., 2014). NAC2 participates in the stabilization and processing of the 5′-UTRs of *psbD* (encoding the D2 subunit of the PSII reaction center), MAC1 for *psaC* (encoding the Photosystem I iron–sulfur protein), and MBB1 for *psbB* mRNA (encoding the PSII core antenna CP47). These factors bind to the 5′-UTR end of their target, protecting the transcript from exonucleases degradation. Additionally, MBB1, an orthologue of HCF107 (high chlorophyll fluorescence 107) protein in *A. thaliana*, participates in the processing of the *psbB-psbT-psbH* polycistronic transcript (Felder et al., 2001). Before translation, this mRNA must be divided into smaller transcripts, *psbB-psbT*, and *psbH* (Vaistij et al., 2000b). MBB1 binds to the 5′-UTR of *psbB* and *psbH* and it is speculated that this could recruit an endonuclease or modify the secondary structure of the mRNA exposing the sites for endonuclease cleavage. Once the polycistronic mRNA is cleaved, MBB1 stabilizes the monocistronic transcripts, protecting them from exonucleases (Loizeau et al., 2014).

## Octotricopeptide Repeat Proteins

Octo-tricopeptide repeat proteins are the most abundant helical repeat proteins in green algae. Their structure is characterized by the presence of motifs of 38–40 amino acids repeated in tandem, in the same way as in PPR and TPR proteins, where each motif forms a pair of antiparallel α-helices (Eberhard et al., 2011; Wang et al., 2015). Besides green algae, OPR proteins are present in some pathogenic bacteria (*Coxiella burnetii*; Rahire et al., 2012), symbiotic protozoa (e. g. *Plasmodium falciparum*, and *Toxoplasma gondii*; Hillebrand et al., 2018; Hollin et al., 2021) and the cercozoan amoeba *Paulinella chromatophora* (Oberleitner et al., 2020); but are rare in land plants. In *A. thaliana*, the presence of only one OPR protein has been reported, called RAP, which is involved in the processing of the 16S ribosomal RNA chloroplast gene (Kleinknecht et al., 2014). In contrast, analysis *in silico* has revealed that there are more than 120 members (Goodstein et al., 2012; Boulouis et al., 2015). The abundance of OPR proteins in the nuclear genome of Chlamydomonas shows not only a high prevalence but also a complex regulation of chloroplast gene expression, where these factors play an essential role. In *C. reinhardtii*, this family of proteins is extensively involved in the post-transcriptional control of chloroplast gene expression (Table 3). The first OPR described in *C. reinhardtii* was the chloroplast translation factor TBC2 (Auchincloss et al., 2002), which together with the nuclear proteins TBC1 and TBC3 interact with the 5′-UTR end of the *psbC* mRNA favoring its translation (Zerges et al., 2003). Like TBC2, TAB1 (Stampacchia et al., 1997; Rahire et al., 2012), TDA1 (Eberhard et al., 2011), and TAA1 (Lefebvre-Legendre et al., 2015; Reifschneider et al., 2016) also function as translation factors in the translation of *psaB*, *atpA*, and *psaA* mRNAs, respectively.

**Table 3:** Octo-tricopeptide repeat proteins identified and studied in *C. reinhardtii*.

Type of protein	Name	Number of OPR motifs	Target	Function	Reference
OPR	CCS2	10	*Possibly ccsA*	Involved in assembly of cytochrome *c* complex.	Cline et al., 2017
	MBC1 (OPR56)	ND	*psbC*	Stabilization of *psbC* mRNA	Cavaiuolo et al., 2017
	MBI1	17	*psbI*	Stabilization of *psbI* 5'UTR	Wang et al., 2015
	MCG1	12	*petG*	Stabilization of *petG* 5'UTR	Wang et al., 2015
	MCD1	7	*petD*	Stabilization of the *petD* 5'UTR	Murakami et al., 2005
	MDA1	13	*atpA*	Stabilization and processing of the *atpA* transcript.	Drapier et al., 1998, 2002; Viola et al., 2019
	MTHI1	9	*atpH, atpI*	Stabilization and translation of *atpH* mRNA. Translation activation and stabilization *atpI* mRNA.	Ozawa et al., 2020
	RAA1	5	*psaA*	*Trans-*splicing of *psaA* mRNA	Rochaix et al., 2004; Merendino et al., 2006; Lefebvre-Legendre et al., 2016
	RAA3	5	*psaA*	*Trans*-splicing of *psaA* mRNA	Rivier et al., 2001; Reifschneider et al., 2016
	RAA8 (OPR120)	6	*psaA*	*Trans*-splicing of *psaA* mRNA	Marx et al., 2015; Lefebvre-Legendre et al., 2016; Reifschneider et al., 2016
	RAT2	2	*psaA*	*Trans*-splicing of *psaA* mRNA and processing of 3' end of *tscA* RNA	Eberhard et al., 2011; Marx et al., 2015; Lefebvre-Legendre et al., 2016; Reifschneider et al., 2016
	TAA1	7	*psaA*	Translation and stabilization of *psaA* mRNA.	Lefebvre-Legendre et al., 2015; Reifschneider et al., 2016
	TAB1	10	*psaB*	Translation of the *psaB mRNA*.	Stampacchia et al., 1997; Wostrikoff et al., 2004; Rahire et al., 2012
	TBC2	9	*psbC*	Translation of *psbC* mRNA	Auchincloss et al., 2002; Zerges et al., 2003; Rahim et al., 2016
	TDA1	8	*atpA*	Translation of *atpA* mRNA	Eberhard et al., 2011; Viola et al., 2019
	OPR1 (Cre01.g001501)	ND	*psaA*	*Trans-*splicing of *psaA* mRNA	Reifschneider et al., 2016
	OPR104 (Cre17.g698750)	ND	*psaA*	*Trans-*splicing of *psaA* mRNA	Reifschneider et al., 2016
OPR-NCL	NCC1	9	*atpA*	Destabilization of *atpA* transcripts	Boulouis et al., 2015
	NCC2	9	*petA*	Destabilization of *petA* transcripts	Boulouis et al., 2015

In addition to facilitating the translation of specific mRNAs, some OPR proteins can perform additional functions or even carry out dual functions (Eberhard et al., 2011; Ozawa et al., 2020). They can function as **M** (maturation) factors, protecting their mRNA target from degradation by exonucleases and/or participate in the processing of polycistronic mRNAs (Murakami et al., 2005; Wang et al., 2015; Viola et al., 2019; Ozawa et al., 2020), as well as acting as factors that participate in gene *trans-*splicing (Reifschneider et al., 2016). The coordinated interaction of these factors on maturation/stability and translation greatly affects organelle function and, consequently, photosynthesis, respiration, and environmental responses. Some of the OPR proteins functionally characterized as **M** factors comprise the proteins MBI1, MCG1, MTHI1, and MDA1, which stabilize the *psbI*, *petG, atpH/atpI*, *and atpA* transcripts, respectively (Wang et al., 2015; Viola et al., 2019; Ozawa et al., 2020). The binding of these proteins to the 5′ UTR protects transcripts against 5′-3′ exonucleolytic degradation by RNA exonucleases.

Interestingly, OPR proteins may also play a double role; in some cases, acting as T factors while also participating in the stabilization of their target transcript. The opposite may also take place, **M** factors contributing to translation of their target. Such is the case of TAA1 (Lefebvre-Legendre et al., 2015) a translation factor required for PsaA accumulation that also participates in preventing *psaA* degradation by exonucleases. Another example of a dual role is the MTHI1 protein; which, unlike TAA1, is involved in regulation of the expression of two different targets, *atpH* and *atpI* mRNAs (Ozawa et al., 2020). On the one hand, MTHI1 works as **M** factor for the *atpH* mRNA, allowing for the accumulation of a monocistronic transcript but also participating as translational enhancer. On the other hand, MTHI1 is required for translation activation of the *atpI* mRNA but not for its stabilization (Ozawa et al., 2020). Conversely, the expression of a single transcript can be regulated by more than one OPR protein; such is the case for the *atpA* transcript in the *atpA*-*psbI*-*cemA*-*atpH* gene cluster (Drapier et al., 1998). The accumulation of the AtpA protein depends on the maturation and translation activities of the MDA1 and TDA1 factors, respectively, both of which bind to the 5′-UTR of *atpA*. While MDA1 stabilizes and participates in processing the *atpA* transcript (Viola et al., 2019), TDA1 intervenes, facilitating the start of translation (Eberhard et al., 2011).

Another OPR protein is CCS2 which is required for the assembly of the cytochrome *c* complex. CCS2 contains an LWALAR consensus amino acids motif (Cline et al., 2017), which is different to the PPPEW motif, typical of other known OPR proteins such as TBC2, TDA1, and TAB1 (Auchincloss et al., 2002; Eberhard et al., 2011; Rahire et al., 2012). Due to its OPR motifs, CCS2 was initially suggested to participate in the maturation, stabilization, or translation of a chloroplast transcript involved in cytochrome *c* assembly. The transcript of the *ccsA* gene (a heme delivery factor required for cytochrome *c* maturation) has been suggested as the CCS2 target. Experimental evidence has shown that CCS2 is not required for stabilization of *ccsA* thus its role as a **M** factor can be discarded. However, CCS2 acting as **T** factor is a matter of consideration. An additional proposed mode of action, although less plausible, is that CCS2 could stabilize the CCS complex (composed of CCS1 and CcsA) *via* protein–protein interactions or by facilitating the recruitment of additional components to this complex (Cline et al., 2017).

Another role attributed to OPR proteins is participation in splicing processes. A clear example of this is the maturation of *psaA* mRNA, which requires two steps of *trans-*splicing (Herrin and Schmidt, 1988; Figure 1B). The *psaA* gene is encoded in three separate exons that are scattered throughout the chloroplast genome. Each of the exons is transcribed separately and has flanking sequences that allow its assembly by forming structures belonging to the group II introns (Kück et al., 1987; Choquet et al., 1988). Additionally, the *trans-*splicing chloroplast A (*tscA*) locus codes for a short non-coding RNA that completes the structure of the first tripartite group II intron (Goldschmidt-Clermont et al., 1991). At least 14 factors encoded in the nucleus participate in the splicing of *psaA* (Reifschneider et al., 2016). Although not all of them have been characterized, at least six of these factors contain OPR motifs: RAT2, RAA1, RAA3, RAA8 (also known as OPR120), OPR1 (Cre01.g001501), and OPR104 (Cre17.g698750; Merendino et al., 2006; Marx et al., 2015; Lefebvre-Legendre et al., 2016; Reifschneider et al., 2016; Figure 1B). All these factors are part of a post-transcriptional complex, presumably functioning as RNA interaction module (Lefebvre-Legendre et al., 2016; Reifschneider et al., 2016; Kück and Schmitt, 2021). On the one hand, RAT2 (RNA-maturation of *psaA-tscA*) is necessary to process *tscA* from a polycistronic precursor (Balczun et al., 2005). While RAA8 (RNA-maturation of *psaA*) participates in the splice junction of the first *psaA* intron (Marx et al., 2015), RAA1 (RNA-maturation of *psaA1*) is involved in the processing of the *tscA* mRNA and participates in the *trans-*splicing of introns 1 and 2 (Rochaix et al., 2004; Merendino et al., 2006). Like many of the nucleus-encoded proteins involved in the anterograde regulation, OPR proteins activity is regulated in response to environmental factors such as light induction (RAA1), light repression (RAA3), H_2_O_2_ suppression (RAA1; Wang et al., 2015), proteolytic degradation in response to nitrogen (MCA1 and TCA1; Wei et al., 2014), and iron deprivation (MAC1 and TAA1; Lefebvre-Legendre et al., 2015; Douchi et al., 2016).

Most functionally known OPR proteins have been identified by screening nuclear mutants with photosynthetic or respiratory defects after mutagenesis. However, Boulouis et al. (2015) characterized two nuclear spontaneous mutants with single amino acid substitutions in two distinct genes encoding OPR proteins: OPR87 (Cre15.g638950.t1) and OPR98 (Cre15.g640400.t1). These nuclear mutants, called *ncc1* and *ncc2*, have expanded the recognition capacity of the OPR proteins, targeting the coding regions of two chloroplast transcripts, leading to the destabilization of *atpA* or *petA* transcripts, respectively (Drapier et al., 2002; Boulouis et al., 2015). NCC1 and NCC2 may recruit an endonuclease or may themselves carry on endo-nucleolytic activity and degrade their new targets (Boulouis et al., 2015). These proteins were classified as NCL (NCC-Like) proteins, a new subfamily of paralogs encoding OPR-RAP proteins, that comprise a highly conserved central region, containing 7–12 OPR repeats and a C-terminal RAP domain (Boulouis et al., 2015). The deeper we study these mutants and other OPR, PPR, and TPR proteins the better we will understand the importance and influence of anterograde control in organellar gene expression regulation. From a biotechnological point of view, elucidating how these proteins bind to nucleic acid sequences and understanding how changes in their motifs, as in the case of NCC1 and NCC2, alter their specificity will have an impact in the development of molecular tools. This will also allow us to skillfully tune the regulation of chloroplast metabolism to obtain new products, change the balance of chloroplast metabolites, and perhaps even achieve the long-sought improvement of photosynthesis.

## Biotechnological Applications of PPR, TPR, and OPR Proteins

The study of PPR proteins in plants has allowed us to elucidate the mechanism by which these proteins recognize and bind to their target mRNA. The level of details is such that it has been possible to postulate an RNA recognition code for PPR proteins (Barkan et al., 2012; Yagi et al., 2013, 2014). Considering that each PPR motif recognizes a ribonucleotide, it has been proposed that the amino acid residues at positions 1, 4, and ii (the penultimate residue) of each PPR motif are determinants for the recognition of their RNA bases. Being the residues 4 and ii, those with the greatest influence on ribonucleotide recognition (Barkan et al., 2012; Yagi et al., 2013; Figures 2C,D). Some of the recognition codes that have been proposed include the amino acids Val, Asn, and Ser at positions 1, 4, and ii, respectively, for cytosine recognition; Val, Asn, and Asp for uracil; Val, Thr, and Asp for guanine; and finally, Phe, Thr, and Asn for adenine (Barkan et al., 2012; Yagi and Shiina, 2014; Figure 2D). However, the bioinformatic analysis predicts that there are other combinations of the PPR code amino acids; in fact, a few of them have been explored (Barkan et al., 2012; Yin et al., 2013; Cheng et al., 2016; Yan et al., 2019). From a biotechnological point of view, elucidating the PPR code is an opportunity area for the design of new RNA-binding proteins with a particular motif or additional domains bound to specific targets for the identification or control of mRNA metabolism.

Imai et al. (2018) suggest using PPR motifs as scaffolds to develop designed RNA-binding proteins. The fusion of PPR motifs with other domains could allow the development of new tools for (a) RNA detection *in vivo* by assembling engineered-PPR (ePPR) to a reporter protein (e.g., GFP); (b) translation regulation by fusing ePPR motif to eIF4G (eukaryotic translation initiation factor 4G), allowing activation translation of the targeted mRNA; (c) development of a site-specific endonuclease, by fusing ePPR motif with an endonuclease, which allows recognizing and cleaving RNA in a specific sequence; (d) RNA editing, using an ePPR protein with cytidine deaminase domains to catalyze cytosine’s reaction to uracil at a specific position; and (e) finally, the use of engineered motifs targeted to otherwise naked or susceptible to degradation mRNA. This, depending on the nature of each original P/O/TPR protein, could be used to increase the half-life of the transcript, enhance translation, or even tune down transcript translatability. The use of designed PPR proteins has great potential in developing tools that require RNA binding or tagging applications. McDermott et al. (2019) recently demonstrated that an artificial protein constructed with PPR motifs targeting a specific RNA-target could specifically bind to it *in vivo* and have low levels of off-target bindings. This work was the first approach to developing engineered PPR proteins as tools to study RNA metabolism in plants.

**Figure 3:**
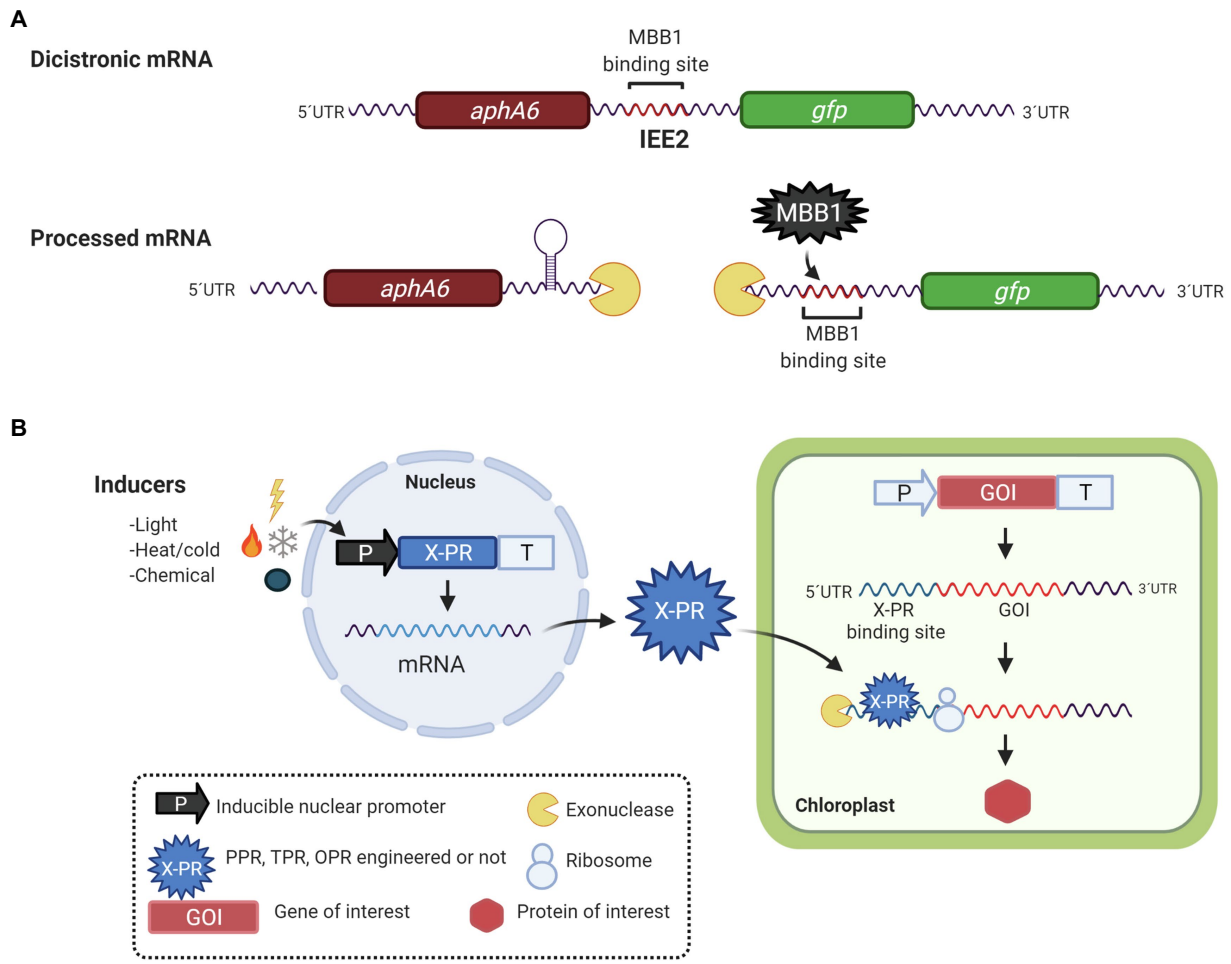
Biotechnological applications of PPR, OPR, and HAT proteins. **(A)** Expression of synthetic operons in *C. reinhardtii*. Binding sites for PPR and HAT-TPR protein have been used as intercistronic expression elements (IEE) for the simultaneous expression of two or more foreign genes. In this example, the IEE2, deriving from the *psbN-psbH* intergenic region, contains a binding site for MBB1, a HAT-TPR protein. When IEE2 was used between two foreign genes, *aphA-6* (conferring resistance to kanamycin) and *gfp* it was suggested that IEE2 acts as a binding site for an M factor thus stabilizing and protecting the *gfp* mRNA from exonuclease degradation and allowing the production of GFP protein. **(B)** Nuclear vector for inducible expression of chloroplast genes. The transcription of a nucleus-encoded helical repeat protein (X-PR, engineered or not) is regulated by an inducible promoter activated by light, temperature (hot or cold), or a chemical inductor. After the X-PR is translated in the cytosol, it is transported to the chloroplast where it anchors to its chloroplast mRNA target. X-PR acts then as M and/or T factor, promoting the production of the protein of interest in the chloroplast.

Another opportunity for the biotechnological applications of PPR, TPR, and OPR proteins is their binding sites. Considering that natively these proteins bind to 5′- and 3′-UTR targets and function as **M** or **T** mRNA factors, it has been proposed to use them in the maturation-stabilization and translation of foreign genes with 5′-UTRs containing P/T/OPR proteins binding sites. Zhou et al. (2007), Legen et al. (2018), and Macedo-Osorio et al. (2018) have identified sequences called Intercistronic Expression Elements (IEEs), which contain TPR and PPR proteins binding sites, that have been used for the expression of heterologous polycistronic mRNA in tobacco (Zhou et al., 2007; Lu et al., 2013; Fuentes et al., 2016; Legen et al., 2018), tomato (Lu et al., 2013), and *C. reinhardtii* (Macedo-Osorio et al., 2018). IEEs were first described by Zhou et al. (2007), who identified a sequence of 50 bases on the 5′-UTR of the *psbH* gene in tobacco. It contains the binding site of the HAT protein HCF107; this element was recognized as an IEE. Under native conditions, HCF107 binds to the 5′-UTR of *psbH* and stabilizes the monocistronic transcript of *psbH* once it is cleaved from the bicistronic transcript *psbT-psbH* (Felder et al., 2001; Sane et al., 2005). When the IEE is inserted into the intergenic region of the bicistronic transcript *yfp-nptII* (yellow fluorescent protein and kanamycin resistance gene, respectively), it is able to stabilize and increase the translation of the monocistronic transcripts (Zhou et al., 2007). Its function has been attributed to the fact that it contains the binding site for HCF107 (Hammani et al., 2012). As expected, HCF107 joins to the 5′-UTR of the foreign gene, stabilizing and protecting the transcript from exonucleases and generating higher translated product (Zhou et al., 2007; Lu et al., 2013; Fuentes et al., 2016). This IEE has been successfully used to express genes involved in the tocochromanol biosynthesis pathway to produce vitamin E in tobacco and tomato (Lu et al., 2013) and for the expression of the core biosynthetic pathway of artemisinic acid production in tobacco chloroplasts (Fuentes et al., 2016). Although, these elements have great potential in metabolic pathway engineering, Legen et al. (2018) recently, demonstrated that when an IEE is used in the stabilization of foreign genes, there is a negative effect on the stabilization of the endogenous *psbH* transcript also, possibly due to a phenomenon of HCF107 titration for competition between the binding sites of the foreign transcript and the native *psbH* transcript. This problem can be solved by designing PPR and TPR proteins with specific binding sites to foreign genes. Additionally, Legen et al. (2018) showed that bindings sites for PPR10, HCF152, and CCR2 can be used as IEEs when they were evaluated in a synthetic *neo-egfp* bicistron in tobacco chloroplasts. Similar results were obtained by Macedo-Osorio et al. (2018) in *C. reinhardtii* when the intergenic region of *psbN-psbH* was used as IEE, which contains the anchoring site for the MBB1 protein, an ortholog of HCF107. This region, called IEE2, could stabilize the monocistronic transcript *gfp* on the bicistronic *aphA6-gfp* mRNA, allowing the translation and accumulation of the GFP protein. Similarly, in tobacco with HCF107, MBB1 could be binding to IEE2, functioning as an M-factor for the GFP monocistronic transcript in *C. reinhardtii* (Figure 3A).

Additionally, Rojas et al. (2019) developed an inducible switch to activate chloroplast transgenes’ expression in tobacco using an engineered PPR proteins. In native conditions, PPR10 binds to the 5′ end of the *atpH* mRNA protecting it from degradation by exonucleases and increasing its translational efficiency. In this case, Rojas et al. (2019) generated variants of PPR10 from maize, with modified amino acids that allow it to bind to a specific target sequence; the protein was expressed in the nucleus under the regulation of an inducible promoter. The protein once produced could bind to a specific target sequence, located in 5′-UTR of the GFP mRNA expressed in the chloroplast. The inducible system allows the regulated expression of chloroplast genes from the nucleus (Figure 3B).

Similarly, Carrera-Pacheco et al. (2020) developed an inducible gene expression system in the Chlamydomonas chloroplast. The OPR protein, TDA1 of Chlamydomonas was nuclear expressed under the regulation of light, and heat-inducible promoters, HSP70A-RBCS2, the generated protein is capable of specifically binding to the 5′-UTR of *atpA* used to regulate the expression of *gfp* in the chloroplast, showing a 1.9-fold increase in the production of the recombinant protein. A reduced number of examples, albeit clear and concise, have shown that it is possible to engineer and manipulate PPR proteins to modulate and rewire gene expression, particularly with a biotechnological application, that is, to express foreign proteins in the chloroplast of plants. Application of engineered PPR, OPR, and TPR proteins in the chloroplast of *C. reinhardtii* has yet to see the light.

## Final Remarks

Penta-tricopeptide repeat, TPR, and OPR proteins have been revealed as essential factors for the functioning of chloroplasts; they act as regulatory factors for maturation, stabilization, and translation of different transcripts coding for essential genes including those for photosynthesis. Although initial studies of PPR proteins in plants allowed the elucidation of their mechanisms of action, it is necessary to characterize additional PPR, TPR, and OPR proteins from *C. reinhardtii* to gain knowledge in their regulatory mechanisms in this important model organism. Understanding the way they control and regulate the expression of genes, mainly in the chloroplast, opens the door to generate new tools to improve not only the production levels of recombinant proteins and food and relevant pharmaceutical compounds but also to perform metabolic engineering aimed at enhancing the production of lipids for biofuels. A more complex task, though not unrealistic would be to remodel cell metabolism in Chlamydomonas to enhance photosynthesis to make the CO_2_ fixation rate more efficient and thus contribute to the reduction of atmospheric CO_2_ levels, while producing sustainable value-added products.

## Author Contributions

KM-O and JB-C: conceptualization. KM-O: investigation and writing of original draft. KM-O, AM-A, and JB-C: writing- reviewing and editing. AM-A: funding acquisition. All authors contributed to the article and approved the submitted version.

## Funding

KM-O was financially supported by CONACYT through a postdoctoral fellowship. Research in the laboratory of JB-C is financially supported by Instituto Politécnico Nacional and CONACYT through project 286497.

## Conflict of Interest

The authors declare that the research was conducted in the absence of any commercial or financial relationships that could be construed as a potential conflict of interest.

## Publisher’s Note

All claims expressed in this article are solely those of the authors and do not necessarily represent those of their affiliated organizations, or those of the publisher, the editors and the reviewers. Any product that may be evaluated in this article, or claim that may be made by its manufacturer, is not guaranteed or endorsed by the publisher.

## Footnotes

^1^ https://ppr.plantenergy.uwa.edu.au/ppr/
